# Beef Quality Assurance (BQA) certification is associated with improved condition, mobility, carcass metrics, and economics in cull dairy cows

**DOI:** 10.3168/jdsc.2025-0979

**Published:** 2026-02-19

**Authors:** Sara McNichols, Ingrid L.B. Fernandes, Nathan E. Rehder, Rebecca L. Weir, Tara L. Felix

**Affiliations:** 1 Department of Animal Sciences, Pennsylvania State University, University Park, PA 16802; 2 Department of Agricultural Economics, Sociology, and Education, Pennsylvania State University, University Park, PA 16802

## Abstract

The objective of this study was to evaluate the effects of Beef Quality Assurance (BQA) certification on relevant traits of cull dairy cows. This study compared BCS, lameness scores, hot carcass weight, and carcass damage of cull dairy cows originating from BQA and non-BQA certified farm operations at the slaughter facility. A blinded observational study was completed to visually score 611 cows from 15 different dairy operations upon arrival at the holding pens of a slaughter facility. Of the 15 farms, 9 were BQA certified. Dairy cows from BQA-certified operations had improved BCS, were less lame, had greater hot carcass weight (346 vs. 283 kg), and had less carcass damage when compared with dairy cows from noncertified farms. If we assume that the difference in hot carcass weight and damage loss can be fully attributed to BQA certification, then the certification led to an additional value of US$251/cow. These findings indicate that BQA certification is associated with improved BCS, mobility, carcass outcomes, and profit potential in cull dairy cows.

## Highlights

• BQA-certified dairies sold cows with better BCS than non-BQA-certified dairies.
• BQA-certified dairies sold cows that were less lame than non-BQA-certified dairies.
• Carcasses were heavier from BQA-certified versus non-BQA-certified dairies.
• Carcasses had fewer defects when from BQA-certified versus non-BQA-certified dairies.
• Heavier hot carcass weight and fewer defects mean that BQA increased cow value by $251/head.

## Graphical Abstract

**Figure:**
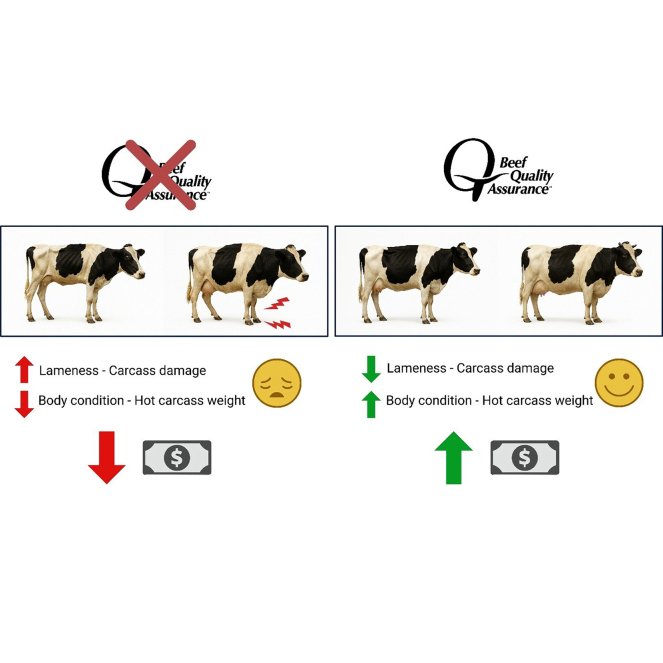
**Summary:** Cattle handling and management techniques are core principles addressed in Beef Quality Assurance (BQA) trainings. To assess the impact of BQA certification on culling decisions of dairy cows, we evaluated cows from farms with and without BQA certification in a blinded observational study. Cows from farms that had BQA-trained employees were culled in better body condition score (BCS) with less lameness and fewer defects in their resultant carcasses compared with those from farms that do not promote BQA certification. These improvements in animal well-being of cull dairy cows resulted in increased value attributed to cows from BQA certified operations when compared with those from non-BQA-certified operations. Created with BioRender.com.

The Beef Quality Assurance (**BQA**) program mission highlights sharing knowledge to ensure producer commitment to food safety, cattle welfare, and beef quality (Beef Quality Assurance, 2025). With both online and in-person training, BQA provides certification to cattle producers that is easy to access. The training focuses on providing science-based knowledge and good husbandry techniques to produce quality beef under ideal management. The acceptance of BQA as the current “gold standard” in training is evidenced by the requirement of this certification for cattle operations desiring to sell in certain markets, and BQA certification is also available to both beef and dairy operations.

The population of beef cows, currently estimated to be 28.7 million head, is larger than that of dairy cows, with only 9.45 million dairy cows (USDA-NASS, 2025). Despite the difference in numbers, in 2023, Zoller (2023) reported that 50% of the cow slaughter in the United States was made up of dairy cows because the dairy sector replaces cows faster than the beef sector. Marketed dairy cows were reported to have more BQA-related defects than beef cows, noted as a greater incidence of both lameness and poor BCS (Ahola et al., 2011).

Adams et al. (2016) conducted an evaluation of the efficacy of BQA training to improve dairy farm worker knowledge. They administered a BQA exam before and after training employees on BQA principles related to handling and management decisions, such as visual evaluation of BCS and lameness. The authors concluded that BQA training could improve dairy farm worker and owner practices related to handling and management of dairy cows based on the knowledge transfer. Furthermore, they suggested that this knowledge transfer had the potential to improve the quality (e.g., BCS, carcass weight) of cull cows being sent to market; however, they did not assess these outcomes directly (Adams et al., 2016). However, other authors (Ahola et al., 2011; Moreira et al., 2021) have reported an association between poor BCS and lameness issues and valuation of cull dairy cows. Thus, the studies highlight the importance of better management to improve dairy producer profit.

Therefore, the objective of this study was to evaluate the effects of BQA certification on BCS, lameness scores, hot carcass weight, and carcass damage of cull dairy cows from BQA-certified and non-BQA certified farm operations at the slaughter facility. We hypothesize that improved culling decisions applied to dairy cows, related to BQA training, could improve the economic value of cows. With minimal research conducted on cull cows in general, the ability to document measurable improvements that may have economic impacts could have industry-wide implications related to cull dairy cows.

A blinded observational study was completed where dairy cull cows (predominantly Holstein) originated from 15 farms, and 9 of those farms were BQA certified (60%). As this was a commercial observation only study, an Institutional Animal Care and Use Committee exemption was granted because researchers simply observed animals moving through a packing plant. Plant buyers sourced cows, ensuring a mix of cows from BQA-certified and non-BQA certified farms, but did not provide this information to evaluators until after BCS and lameness scores were assigned. In total, 611 animals were evaluated; however, not every observed metric was captured by the observers on every cow. Missing observations were due to animal movement, pen access limitations, or unavailable plant records. Therefore, cow numbers reported for each metric are as follows: lameness score (BQA, n = 337; non-BQA, n = 200), BCS (BQA, n = 339; non-BQA, n = 199), hot carcass weight (BQA, n = 345; non-BQA, n = 223), and carcass damage (BQA, n = 356; non-BQA, n = 255). Because culling of the dairy cow herd occurs year round, and with minimal seasonal fluctuations compared with culling of the beef cow herd, 2 representative months (October 2024 and June 2025) were chosen for data collection in the Northeastern United States.

Cull cows were hauled from their farm of origin to the commercial facility and unloaded into lairage. Once in lairage, an interobserver agreement of at least 60% was established, and then cows were assessed by 4 evaluators to assign both lameness scores and BCS.

As described by Sprecher et al. (1997), lameness was evaluated on a scale from 1 to 5, where 1 = normal stride and 5 = severely lame (Sprecher et al., 1997; Thomsen et al., 2008). Similarly, BCS was evaluated from 1 to 5 (Wildman et al., 1982), where 1 = emaciated and 5 = obese, with 3 being average, or “ideal,” for most lactating dairy cows (Roche et al., 2009).

Carcass trait data, including hot carcass weight and carcass damage, or defects, and were provided by the plant. The hot carcass weight is the weight of the carcass of an animal after removal of the head, hide, internal organs, and gastrointestinal tract but before being chilled (Troxel and Gadberry, 2015). Carcass damage included carcass weight loss in any one of the following categories: light or heavy trim; arthritic stiff joints; or necessary trimming of the skirt, legs, briskets, or rounds. In this particular study, the purpose was not to identify the types of defects but rather simply to quantify damage to the carcasses. Therefore, carcass damage was defined as any incidence of damage to the carcass (binomial distribution; 1 = carcass damage and 0 = no carcass damage). Carcasses with more extensive damage were segregated in the analysis to account for any that had damage in more than one of the previously defined carcass damage categories.

Finally, to attribute economic contributions to BQA certification, weekly price data for carcasses 500 lb (227 kg) and heavier was collected from the USDA Agricultural Marketing Service from January 3, 2020, to October 10, 2025. Because sale price was not known, cows were not sorted into categories (e.g., boner, breaker) rather, as stated, the analysis simply represents the average dressed-domestic, cutter (90% lean) price for carcasses equal to or greater than 500 lb (227 kg). The maximum price during this period was $6.96/kg, and the minimum price was $2.35/kg, with an average price of $4.07/kg (USDA-AMS, 2025). Therefore, the price we used in the analysis was an average of the prices observed from this time and did not consider breaker (75% lean) and boner (85% lean) prices in this analysis, which may underestimate economic differences.

Once all data were collected, the MIXED procedure of SAS (Version 9.4; SAS Institute, 2013) was used to analyze the impact of BQA certification on hot carcass weight and average carcass price, with BQA status (certified or not) and period (June or October) as fixed effects. Period was included as a fixed effect in the model because it was significant and provided the model of best fit based on the Bayesian information criterion. Farm nested within BQA status was included in the model as a random effect to address clustering.

The GLIMMIX procedure of SAS was used to determine the effect of farm classification (BQA certified or non-BQA certified) on the incidence of BCS (redefined as BCS 1 or 2 = thin, BCS 3 = ideal, and BCS 4 or 5 = fat), lameness score (1 through 5), carcass damage, and additional carcass damage. Farm classification was a fixed effect in the model. Data were analyzed via binomial distribution with a Satterthwaite adjustment for degrees of freedom. Period was not included in the model because it was not significant and did not improve the Bayesian information criterion.

Two GLIMMIX procedures with logit link models were used to determine the likelihood of a non-BQA certified farm having (1) thin, as defined previously, or (2) severely lame cows (redefined as lameness score 1 = not lame, lameness score 2 and 3 = moderate lame, and lameness score 4 or 5 = severely lame). Fixed effects included were BQA certification status and period. Producer farm was included as a random intercept to account for clustering of cows within BQA status. The model was fit using Laplace approximation, and results are reported as odds ratios with corresponding confidence intervals. Cow was the experimental unit in all models, and significance was declared at *P* < 0.05.

Cows sourced from non-BQA certified farms had 9.89-fold greater odds (95% CI: 2.721–35.981; *P* = 0.0005) of being severely lame (lameness score 4 and 5). The proportion of animals that the industry would classify as not lame (score 1) was greater in cows sourced from BQA certified farms (43.9%; 148/337) when compared with cows sourced from non-BQA certified farms (32.5%; 65/200; Figure 1). Moreira et al. (2021) reported that foot and leg problems were the most financially detrimental culling reasons in dairy operations, indicating that severely lame cows could incur large discounts.

**Figure 1:**
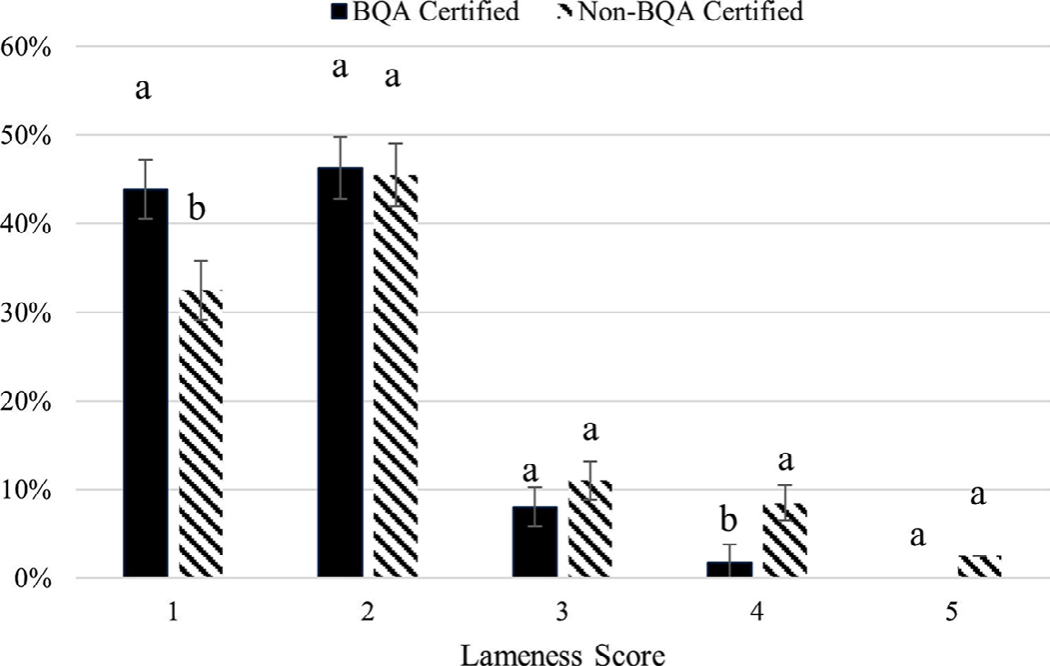
The comparison of lameness scores between cull dairy cows at the slaughter facility originating from Beef Quality Assurance (BQA) certified farms (solid bar; n = 337) and non-BQA certified farms (hashed bar; n = 200). The proportion of animals classified as severely lame (score 4 and 5) was greater in cows sourced from non-BQA certified farms when compared with cows sourced from BQA-certified farms (*P* < 0.05). Lameness score means with different lowercase letters are different (*P* < 0.05). The figure displays evidence that BQA certification was associated with fewer cows culled when they were severely lame. Error bars depicted are associated with the SEM (SEM = 1.97).

Cows sourced from non-BQA operations had 4.33-fold greater odds (95% CI: 2.124–8.831; *P* < 0.0001) of having a BCS of 1 or 2, which the industry would classify as “thin” (Figure 2). Dairy farms use BCS as a practical and noninvasive way to monitor the nutrition and health status of dairy cows. Having a BCS too low (≤2) or high (>3) can be a concern for the health and well-being of the cow, resulting in challenges to reproductive and productive efficiency (Heinrichs et al., 2023). Dairy cows with reduced BCS (i.e., “thin cows”) are associated with an increased incidence of illness (Roche et al., 2009), poor nutrition and management, or other issues that ultimately affect culling decisions. Sánchez-Hidalgo et al. (2020) suggested that the condition of an animal at slaughter directly correlated with the welfare of the animal's herd of origin. Corroborating these findings, thin cows were associated with greater incidence of lameness, bruising, and injury (Ahola et al., 2011; Moreira et al., 2021). These challenges in thin cull cows could result in large discounts in the live price due to postslaughter trimming and additional weight losses; however, price differences related to condition were not explicitly assessed in this study.

**Figure 2:**
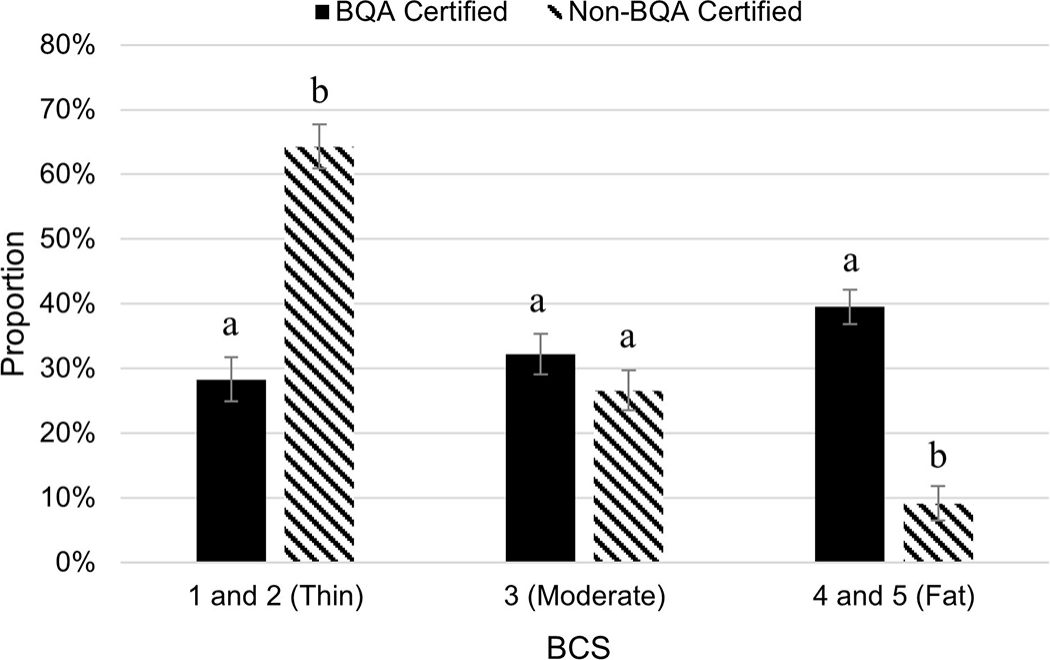
The comparison of BCS between cull dairy cows at the slaughter facility originating from Beef Quality Assurance (BQA) certified farms (solid bar; n = 339) and non-BQA certified farms (hashed bar; n = 199). The proportion of animals classified as thin (score 1 or 2) was greater when cows were sourced from non-BQA certified farms when compared with cows sourced from BQA-certified farms (*P* < 0.05). Body condition score means with different lowercase letters are different (*P* < 0.05). The figure displays evidence that BQA certification was associated with culling fewer thin cows and more cows in fat condition. Error bars depicted are associated with the SEM (SEM = 3.4).

Cows sourced from BQA-certified operations had hot carcass weights that were 63 kg greater than cows sourced from non-BQA certified farms (*P* < 0.01; Table 1). Some of these differences in carcass weight may be attributed to carcass damages, noted by the plant. Of the total carcasses assessed, only 5.6% (20/356) of carcasses from cows originating from BQA-certified farms had at least one type of carcass damage, whereas 18.4% (47/255) of carcasses from cows originating from non-BQA operations had carcass damage (*P* < 0.01). In addition, those carcasses belonging to cows from non-BQA operations tended (*P* = 0.06) to have at least 2 instances of damage more frequently than carcasses from cows from BQA-certified farms (Table 1). Carcass damage can be the consequence of antemortem issues such as lameness and poor BCS, which necessitate carcass trimming and, thus, caused weight loss. Additionally, poor housing conditions, which could cause skin injuries before slaughter, are related to carcass damage (Knock and Carroll, 2019).

**Table 1:** Comparison of carcass metrics between cull dairy cows at the slaughter facility originating from Beef Quality Assurance (BQA) certified farms and non-BQA certified farms

Item	Farm of origin BQA status^1^		SEM	*P*-value
	BQA certified	Non-BQA certified		
Hot carcass weight, kg	346	283	8.63	<0.01
Carcass damage,^2^ % (n/n)	5.6 (20/356)	18.4 (47/255)	2.43	<0.01
Additional carcass damage,^3^ % (n/n)	4.5 (16/356)	8.2 (20/255)	1.72	0.06
Carcass value,^4^ US$/carcass	1,402	1,151	35.37	<0.01

1 Cull cows at the slaughter facility originated from farms that either completed BQA training (BQA certified) or had not done BQA training (non-BQA certified).

2 Carcass damage categories defined as light or heavy trim; arthritic stiff joint; or damage to the skirt, legs, brisket, or round. Expressed as the percentage of the total carcasses summed across all damage categories.

3 Includes any carcasses that had damage in more than one carcass damage category: light or heavy trim; arthritic stiff joints; or damage to skirts, legs, briskets, or rounds.

4 Assuming that the difference in hot carcass weight is fully attributed to BQA certification. Calculated a 5-yr average price using dressed-domestic cutter cow carcasses weighing 500 lb (227 kg) or more from the USDA-Agricultural Marketing Service from January 3, 2020, to October 10, 2025 (USDA-AMS, 2025).

Data analyses were extended by estimating the economic return for BQA certification. To become BQA certified, a producer must participate in both in-class and hands-on training, and each training session is ∼2 h. These are free of charge, so the only investment costs involved in obtaining certification are the time cost for the producer and any employees to attend and participate in the training. The return on investment is the additional value that is derived from BQA certification, which in this study is the additional revenue from each culled cow. As previously stated, these data indicate that cull dairy cows originating from BQA-certified farms had an average hot carcass weight of 346 kg, whereas cull dairy cows originating from non-BQA-certified farms had an average of 283 kg (Table 1). From January 3, 2020, to October 10, 2025, the average dressed price for cull cow carcasses of 227 kg or more was $184.39/45.4 kg, which is equivalent to $4.07/kg, as reported by USDA-AMS (2025). If it is assumed that the difference in hot carcass weight can be fully attributed to BQA certification, then the certification equates to a potential additional value of US$251/head (Table 1). It is important to note that the estimated difference of 63 kg in hot carcass weight for BQA-certified producers is likely biased upward because there is no control for other factors beyond BQA certification that might be driving that difference, and this result should be interpreted cautiously. For example, cull dairy cows sold in auction often have a discount due to visual defects such as poor BCS or lameness (Moreira et al., 2021). However, the certification process involves training for best management practices, including feeding practices, proper injections, and cattle handling, and these enhanced practices are likely the factors driving the hot carcass weight differences through the certification process, although these data do not allow for decomposition of the association. Lastly, although the certification process is free of charge, there may be additional costs incurred to adopt practices described in the training, and thus the benefit/cost ratio may be less than our estimate. Thus, although current market prices, unobserved additional costs to implement practices, and weight differences directly attributable to certification all influence the economic return, based on the observable data, there is a positive return for investment in BQA certification.

In conclusion, this study evaluated the effects of BQA certification on welfare and carcass traits, comparing BCS, lameness scores, hot carcass weight, and carcass damage of cull dairy cows originating from BQA-certified and non-BQA certified herds. These results implied the advantage in cull cow carcass value due to BQA certification will be realized if these cows are sold into a carcass quality and quantity pricing system. Partial realization of this financial advantage may be realized via sale of these cows on a liveweight and BCS basis. For example, cows with above-threshold BCS could bring a greater live price. However, the accuracy of accounting for carcass damage categorical losses in live cow pricing is unclear. Because most cull Holstein cows are sold via auction markets, the realization of the BQA certification advantage may be incomplete. Further exploration of marketing methods whereby there can be full realization of the BQA certification advantage is needed.
